# Molecular Epidemiology of *P*. *vivax* in Iran: High Diversity and Complex Sub-Structure Using Neutral Markers, but No Evidence of Y976F Mutation at *pvmdr1*

**DOI:** 10.1371/journal.pone.0166124

**Published:** 2016-11-09

**Authors:** Yaghoob Hamedi, Khojasteh Sharifi-Sarasiabi, Farzaneh Dehghan, Reza Safari, Sheren To, Irene Handayuni, Hidayat Trimarsanto, Ric N. Price, Sarah Auburn

**Affiliations:** 1 Infectious and Tropical Diseases Research Center, Hormozgan University of Medical Sciences, Bandar Abbas, Hormozgan Province, Iran; 2 Molecular Medicine Research Center, Hormozgan University of Medical Sciences, Bandar Abbas, Hormozgan Province, Iran; 3 Hormozgan University of Medical Sciences, Bandar Abbas, Hormozgan Province, Iran; 4 Global and Tropical Health Division, Menzies School of Health Research and Charles Darwin University, Darwin, Northern Territory, Australia; 5 Bioinformatics Laboratory, Eijkman Institute for Molecular Biology, Jakarta, Indonesia; 6 The Ministry of Research and Technology (RISTEK), Jakarta, Indonesia; 7 Agency for Assessment and Application of Technology, Jakarta, Indonesia; 8 Centre for Tropical Medicine and Global Health, Nuffield Department of Clinical Medicine, University of Oxford, Oxford, United Kingdom; Centro de Pesquisas Rene Rachou, BRAZIL

## Abstract

**Background:**

Malaria remains endemic at low levels in the south-eastern provinces of Iran bordering Afghanistan and Pakistan, with the majority of cases attributable to *P*. *vivax*. The national guidelines recommend chloroquine (CQ) as blood-stage treatment for uncomplicated *P*. *vivax*, but the large influx of imported cases enhances the risk of introducing CQ resistance (CQR).

**Methodology and Principal Findings:**

The genetic diversity at *pvmdr1*, a putative modulator of CQR, and across nine putatively neutral short tandem repeat (STR) markers were assessed in *P*. *vivax* clinical isolates collected between April 2007 and January 2013 in Hormozgan Province, south-eastern Iran. One hundred blood samples were collected from patients with microscopy-confirmed *P*. *vivax* enrolled at one of five district clinics. In total 73 (73%) were autochthonous cases, 23 (23%) imported cases from Afghanistan or Pakistan, and 4 (4%) with unknown origin. 97% (97/100) isolates carried the F1076L mutation, but none carried the Y976F mutation. STR genotyping was successful in 71 (71%) isolates, including 57(57%) autochthonous and 11 (11%) imported cases. Analysis of population structure revealed 2 major sub-populations, *K*1 and *K*2, with further sub-structure within *K*2. The *K*1 sub-population had markedly lower diversity than *K*2 (*H*_E_ = 0.06 vs *H*_E_ = 0.82) suggesting that the sub-populations were sustained by distinct reservoirs with differing transmission dynamics, possibly reflecting local versus imported/introduced populations. No notable separation was observed between the local and imported cases although the sample size was limited.

**Conclusions:**

The contrasting low versus high diversity in the two sub-populations (*K*1 and *K*2) infers that a combination of local transmission and cross-border malaria from higher transmission regions shape the genetic make-up of the *P*. *vivax* population in south-eastern Iran. There was no molecular evidence of CQR amongst the local or imported cases, but ongoing clinical surveillance is warranted.

## Introduction

Malaria remains an important infectious disease in Iran. National Malaria Control Programs (NMCPs) initiated in the 1950s were successful in reducing malaria cases in the south-eastern provinces and eliminating malaria in the northern Caspian region by 1977 [1–3]. However, by the early 1990s, new cases of malaria were being reported in the north, associated with the large displacement of populations from Armenia and Azerbaijan. The prevalence of malaria also increased in the south-eastern provinces due to high rates of migration across porous borders with Afghanistan and Pakistan [3–6]. The malaria situation in the south-eastern provinces of Sistan-Baluchistan, Hormozgan and Kerman is now hypoendemic, with approximately 2000 cases a year, of which 95% are due to *Plasmodium vivax* [7].

The proportionately high prevalence of *P*. *vivax* infections underlines the challenges of eliminating this highly adaptive species. The reservoir of dormant parasites in the liver (hypnozoites) presents one of the greatest obstacles to efforts to control *P*. *vivax*, compounding the risks of importation and potential of resurgence [8]. Antimalarial drug resistance presents further challenges to the containment and ultimate elimination of malaria in this region. Studies undertaken in regions with high-grade drug resistant *P*. *vivax* infection have reported severe and life-threatening disease particularly in young children and pregnant women [9, 10].

The two current frontline therapies for *P*. *vivax* in Iran include CQ for schizontocidal therapy and primaquine (PQ) for radical cure, introduced in 1946 and 1950, respectively. Reports of declining chloroquine (CQ) efficacy across much of the vivax-endemic world emphasize the urgent need to reduce transmission within and across borders to contain the spread of resistant infections [11]. Although CQ has historically demonstrated high efficacy for the treatment of *vivax* malaria in Iran, recent trials conducted in the south-east of the country suggest early signs of declining drug susceptibility [12, 13]. The molecular mechanisms of CQ resistance (CQR) in *P*. *vivax* remain largely unknown. To date, studies have focused primarily on *pvcrt-o* (*chloroquine resistance transporter*) and *pvmdr1* (*multidrug resistance 1*), orthologues of genes involved in CQR in *P*. *falciparum* [14]. Investigations of single nucleotide polymorphisms (SNPs) in *pvcrt-o* found no association with the *in vivo* clinical response to CQ [15, 16]. Although a Brazilian study of *pvcrt-o* expression found elevated levels of expression in patients with severe disease and poor response to CQ [17], a more recent study from Papua province, Indonesia, where *P*. *vivax* is highly resistant to CQ, found no correlation between *pvcrt-o* expression and the parasite *ex vivo* response to CQ [18].*Pvmdr1* encodes a digestive vacuole membrane transporter, with SNPs associated with *ex vivo* CQ susceptibility [14, 16, 19]. Because CQR has been reported in *P*. *vivax* isolates without *pvmdr1* mutations, this gene appears to be a minor determinant of CQR in *P*. *vivax*, but presents a useful measure of reduced CQ susceptibility [14].

Parasite genotyping has potential to inform on parasite transmission dynamics [20–22]. Indeed, studies of field populations of *P*. *vivax* using neutral markers such as microsatellites have demonstrated that a spectrum of population structures exist for this species [20, 23]. Previous studies in Iran have assessed the diversity of the local *P*. *vivax* population using genes encoding surface proteins such as *ama1 (apical membrane antigen 1)*, *csp* (*circumsporozoite protein*) and *msp1* (*merozoite surface protein 1*) [24, 25]. However, genetic diversity at loci encoding parasite ligands may be affected by selective pressures from the host immune system, confounding assessment of transmission-related dynamics [26]. The current study aimed to characterise the local transmission dynamics and prevalence of common *pvmdr1* mutations in *P*. *vivax* in the south-eastern province of Hormozgan.

## Materials and Methods

### Study Site

**Fig 1:**
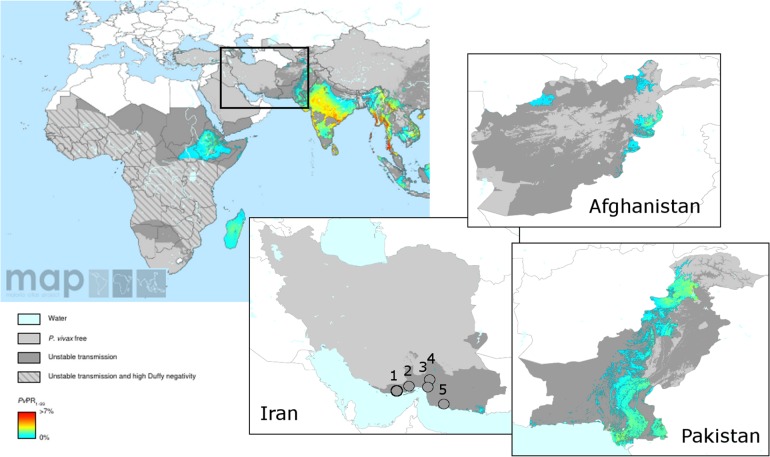
*P*. *vivax* prevalence maps. These maps were generated by the Malaria Atlas Project, University of Oxford. The colour scales reflect the model-based geostatistical point estimates of the annual mean *P*. *vivax* parasite rate in the 1–99 year age range (*Pv*PR_1-99_) [17] within the stable spatial limits of transmission in 2010. The approximate locations of the study sites described here are indicated with numbered open circles in the Iran panel: Bandar Lengeh County (1), Qeshm County (2), Minab County (3), Rudan County (4) and Jask County (5). All MAP maps are available to users under the CCAL 3.0. http://www.map.ox.ac.uk/about-map/open-access/.

The study was conducted in 5 healthcare centers in Hormozgan Province between April 2007 and January 2013. The locations of these sites (Jask, Minab, Bandar Lengeh, Rudan and Qeshm) are shown in Fig 1. Hormozgan Province is located on the northern coast of the Persian Gulf and Gulf of Oman. The region receives a moderate number of migrants from Afghanistan and Pakistan, both of which have endemic *P*. *vivax* (Fig 1). The weather in Hormozgan Province is sufficiently warm and humid to sustain active *Anopheles* populations throughout the year. *An*. *stephensi* is the main malaria vector in the region [27]. Although transmission occurs throughout the year, seasonal patterns are observed with two peaks, the first in April-June and the second in August-October. The national malaria control program has been successful in reducing transmission in recent years, with annual reported cases dropping from 20,000 in 2005 to <2000 in 2012 [28]. Details on the local malaria epidemiology in each of the districts are provided in the Supplementary Material (S1 Table).

### Sample collections

A sample size of 100 was defined to enable detection of common *pvmdr1* mutations in Hormozgan Province. A pilot study revealed that almost all isolates carried the F1076L mutation. Assuming a 90% prevalence of *pvmdr1* mutations in Hormozgan Province, a sample size of 100 would define the true prevalence within +/- 7%. Samples were selected from informed, consenting symptomatic patients attending the clinics who were diagnosed with *P*. *vivax* mono-infection (mixed infections were not enrolled) by microscopic blood film examination and who were willing to donate a venous blood sample. Microscopic examination was undertaken on both thin and thick blood films and the slides were cross-checked by the Quality Control Division. All patients were treated according to the national guidelines with a 3 day course of chloroquine (25 mg/kg) and a 14 day course of primaquine (15 mg). Venous blood samples were collected prior to administration of antimalarial treatment and stored frozen at -20°C until DNA extraction. Details on patient nationality and recent travel history were documented for each patient. Infections in patients who had arrived in Iran within 1 month prior to presenting at the clinic were defined as “imported” cases. A global study of geographic variation in *P*. *vivax* relapse patterns identified a median time to first relapse of 108–120 days (3–4 months) in the ecological zone encompassing Pakistan and southern Afghanistan, and 186–299 days (6–10 months) in the ecological zone encompassing northern Afghanistan [29]. In view of the long duration of relapse patterns in this area and the markedly lower incidence of *P*. *vivax* infection in Iran compared to Afghanistan or Pakistan during the study period (1,418 versus 53,609 and 215,950 *P*. *vivax* cases respectively in 2012 [28], infections in patients who had arrived in Iran between 1 and 10 months prior to presentation at the clinic were also likely to be relapses from infections acquired outside of Iran and were thus defined as “putative imported” cases. All other infections were defined as “autochthonous” cases.

### DNA Extraction and *Pvmdr1* Sequencing

DNA extraction was undertaken on 100 μl whole blood using the DNG-plus kit (CinnaGen, Iran) according to the manufacturer's protocol, and the DNA was stored at -20°C. A nested PCR protocol was used to amplify the *pvmdr1* gene using previously described primers Brega *et al* [17]. For the primary PCR, a ~4392 bp product was amplified using primers *pvmdr1* F1 (5′- TTGAACAAGAAGGGGACGTT-3´) and *pvmdr1* R1 (5′- CTTATATACGCCGTCCTGCAC -3′). For the nested PCR reaction, a ~600 bp fragment was amplified using primers *pvmdr1* F2 (5′- ATAGTCATGCCCCAGGATTG -3´) and *pvmdr1* R2 (5′- CATCAACTTCCCGGCGTAGC -3′). Each of the primary and nested PCR reactions were performed in a 25 μL reaction volume comprising 2 units *Taq* DNA Polymerase, 0.2 μM each primer, 0.2 mM dNTPs, 1X PCR buffer, 3 mM MgCl_2_ and template. The primary reaction used 1 μl genomic DNA as template and the nested PCR reaction used 2 μl primary product diluted 1/50 with PCR-grade water. Thermocycling for the primary reaction was performed under the following conditions: 95°C for 5 min, followed by 35 cycles of denaturation at 94°C for 1 min, annealing 60°C for 1 min, extension at 72°C for 1 min, with a final extension step at 72°C for 10 min. Thermocycling for the nest reaction was performed under the following conditions: 95°C for 5 min, followed by 30 cycles of 94°C for 1 min, 62°C for 1 min, and 72°C for 1 min, with a final extension step at 72°C for 10 min. The PCR products were treated with a pre-sequencing kit (USB Corporation, Cleveland, Ohio) and then sequenced using an Applied Biosystems (ABI) terminator cycle sequencing ready reaction kit (BigDye1 Terminator V3.1 Cycle SequencingKit) on an ABI 3130 genetic analyser by a service provider (Macrogen, South Korea). Sequencing was performed on both strands. The resultant sequences were manually edited and aligned against the Salvador-1 (Sal-1) reference strain (Accession no. AY618622) using GeneRunner software (version 3.05), and all polymorphic positions were recorded. Accession numbers for the *pvmdr1* sequences from Hormozgan Province that were used in the comparison against Sal-1 are as follows; KM216181 (PvYHSHD31J, no mutation); KM496317 (PvYHSHD20J, F1076L mutation); KM216184 (PvYHSHD41P, F1076L mutation and synonymous mutation at T1106 codon); KM216182 (PvYHSHD3J, F1076L and N1010S mutations); KM216183 (PvYHSHD10M, F1076L, L953F and N1010S mutations).

### Single Tandem Repeat (STR) Genotyping

Genotyping was undertaken at nine previously described STR markers: *Pv3*.*27*, *msp1F3*, *MS1*, *MS5*, *MS8*, *MS10*, *MS12*, *MS16* and *MS20* [30, 31]. These markers are included in a consensus panel selected by partners within the Vivax Working Group of the Asia Pacific Malaria Elimination Network (APMEN) [32]. All markers were amplified in nest or semi-nest reactions, with a 20 μl volume comprising 1.5 units *Taq* DNA polymerase, 0.25 μM each primer, 0.2 mM dNTPs, 1X PCR buffer, 2 mM MgCl2, and 1 μl template (genomic DNA for the primary reaction, and diluted first-round PCR product for the nest reaction). Primer details for each of the markers are presented in S2 Table. For *MS1*, *MS5*, *MS8*, *MS10*, *MS12* and *MS20*, thermocycling for the primary reactions was undertaken as follows: 94°C for 3:30 min, followed by 30 cycles of 94°C for 30 s, 58°C for 40 s, and 72°C for 30 s, with a final step of 72°C for 5 min. Thermocycling for the nest reactions was undertaken using the same cycling conditions with the exception that only 25 cycles were performed. For *Pv3*.*27*, *msp1F3* and *MS16*, thermocycling for the primary reactions was undertaken as follows: 95°C for 5 min, followed by 30 cycles of 95°C for 1 min, X°C for 1 min (where X = 57°C for *MS16*, 58°C for *Pv3*.*27*, and 59°C for *msp1F3*), and 72°C for 1 min, with a final step of 72°C for 5 min. Thermocycling for the nest reactions was undertaken as follows: 95°C for 5 min, followed by 25 cycles of 95°C for 1 min, X°C for 1 min (where X = 56°C for *MS16*, 58°C for *Pv3*.*27*, and 60°C for *msp1F3*), and 72°C for 1 min, with a final step of 72°C for 5 min. Fragment sizing was undertaken on the labelled PCR products using denaturing capillary electrophoresis on an ABI 3100 Genetic Analyzer. GeneScan LIZ-600 (Applied Biosystems) was used as internal size standards. GeneMapper Version 4.0 was employed for genotype calling. An arbitrary fluorescent intensity threshold of 100 relative fluorescence units (rfu) was applied for peak calling to reduce artefacts from background noise. All electropherogram traces were additionally inspected manually to omit any further artefacts such as overlap (bleed) peaks or stutter. Where genotypes presented multiple alleles, only the predominant allele and any additional alleles with minimum 33% height of the predominant allele were scored. As described previously, the 33% minor allele calling threshold was applied to reduce potential artefacts [33].

### STR Population Genetic Analysis

Given that *Plasmodium* parasites are haploid during the blood stage of infection, the presence of multiple alleles at a given locus is indicative of a polyclonal infection. In the current study, infections were defined as polyclonal if multiple alleles were observed at one or more loci. The multiplicity of infection (MOI) for a given sample was defined as the maximum number of alleles observed amongst all loci tested. In order to avoid oversampling rare alleles, with the exception of measures of polyclonality and MOI, only the predominant allele at each locus in each isolate was used for analysis [33]. The expected heterozygosity (*H*_E_) was measured as an index of population diversity. This metric determines the probability that two given samples randomly selected from a population will exhibit different genotypes at the locus/loci in question. Expected heterozygosity was measured for each locus using the formula *H*_E_ = [*n*/ (*n*-1)] [1-Σ*p*_*i*_^2^], where *n* is the number of isolates analyzed and *pi* is the frequency of the *ith* allele in the population. Evidence of population structure was sought using STRUCTURE software version 2.3.3 [34]. STRUCTURE analysis was performed using 20 replicates with 100,000 burn-in and 100,000 post burn-in iterations for each of *K* (populations) from 1–10 using admixture and correlated allele frequency model parameters. The most probable *K* was derived using the *delta K* method [35], with bar plots prepared using *distruct* software version 1.1 [36]. In isolates with no missing data, multi-locus genotypes (MLGs) were reconstructed from the predominant allele at each locus. The MLGs were used to measure multi-locus linkage disequilibrium (LD), applying the standardised index of association (*I*_A_^S^) using the web-based LIAN 3.5 software [37]. The significance of the *I*_A_^S^ estimates was assessed using 10,000 random permutations of the data. LD was assessed in 1) all samples, and 2) in unique MLGs only (i.e. with repeated MLGs represented once). The genetic relatedness between sample pairs was assessed by measuring the proportion of alleles shared (*ps*) between pairs of MLGs. Using (1-*ps*) as a measure of genetic distance [38], an unrooted neighbour-joining tree [39] was generated using the ape package in the R software [40]. The correlation between genetic and temporal distance was assessed using Mantel’s *r*-test with 10,000 permutations using the ade-4 package in the R software [41].

### Statistical Analysis

Statistical comparisons of patient gender proportions and of *pvmdr1* haplotype proportions between sample groups were undertaken using Pearson’s Chi-square test. The significance of difference between sites with regard to patient age was assessed using the Mann-Whitney U test. All tests were performed using R software, with a significance threshold of 0.05.

### Ethical Approval

The study was approved by the Ethics Committee of the Infectious and Tropical Disease Research Center, Hormozgan University of Medical Sciences (HUMS 9014). Written informed consent was obtained from all study participants or a parent or guardian where participants were 18 years of age or younger.

## Results

### Patient Sampling

Table 1 provides patient and parasitological details for the 100 samples included in the study, which comprised microscopy positive *P*. *vivax*-infected blood samples collected from patients attending health centers in Hormozgan Province between April 2007 and January 2013. The majority of patients were Iranian nationals (71%), followed by Pakistani (19%) and Afghani (10%) nationals. A total of 73 (73%) cases were defined as autochthonous, including the cases from all 71 Iranian nationals and 2 Afghani nationals; 23 (23%) cases were defined as imported or putatively imported, including cases from 6 Afghani and 17 Pakistani nationals; 4 (4%) cases were defined as unknown with regards importation owing to missing data, including cases from 2 Afghani and 2 Pakistani nationals. The median age of all patients was 21 years (range: 3–86 years), with no significant difference between autochthonous (median 19.5 years) and imported (median 21 years) cases (*P* = 0.172). The majority (76%) of patients were males and this was most apparent in imported (91%, 21/23) compared to autochthonous (71%, 52/73) cases but, again, the difference was not significant (*P* = 0.092).

**Table 1:** Patient sampling details.

Autochthonous vs Imported	Number ^1^	Collection period	Median age (range), years^2^	% Males	% Patients analyzed ^3^
**Autochthonous**	73	Apr 2007- Aug 2012	19.5 (3–86)	71% (52/73)	78% (57/73)
**Imported**^**4**^	23	Apr 2007 –Jan 2013	21 (13–41)	91% (21/23)	48% (11/23)
**Uncertain**	4	Dec 2007 –Jul 2011	24 (22–27)	75% (3/4)	75% (3/4)
All	100	Apr 2007 –Jan 2013	21 (3–86)	76% (76/100)	71% (71/100)

^1^ Number of microscopy-determined *P*. *vivax* positive patients enrolled in the study.

^2^ Missing data for 3 autochthonous, 2 imported and 1 unknown patient.

^3^ Samples with ≥50% successful genotype calls across the 9 markers.

^4^ Includes 3 putative imported cases from 3 patients who presented at the clinic between 1 and 6 months of returning from travel.

### *Pvmdr1* Polymorphisms

All 100 samples were successfully amplified and sequenced at the *pvmdr1* locus. Although none of the isolates carried the Y976F mutation, 97 (97%) isolates carried the F1076L mutation, which confers a phenylalanine to leucine change at codon 1076 (Table 2). Other mutations observed in the population included a nonsynonymous mutation at codon 953 (phenylalanine substituted by leucine) in 1 (1%) isolate, and a nonsynonymous mutation at codon 1010 (serine substitution by asparagine) in 2 (2%) isolates (Table 2). A synonymous mutation was also observed at codon 1106 (ACC to ACT, both coding for Threonine) in 1 (1%) isolate. All 3 wild-type F1076 variants, as well as the single L953F and 2 N1010S mutations were observed in all Iranian nationals. All 29 (100%) of the isolates from Afghan and Pakistani patients had a single F1076L mutation (Table 2). There was no significant difference in the proportions of the wild type (*P* = 0.632), single mutant (*P* = 0.337), double mutant (*P* = 0.642) or triple mutant (*P* = 0.642) *pvmdr1* haplotypes between the Iranian versus Afghani and Pakistani patients.

**Table 2:** Summary of Pvmdr1 haplotypes.

Haplotype	% all patients	% Iranians	% Afghans and Pakistanis
Wild type: Y976, F1076	3% (3/100)	4.2% (3/71)	0% (0/29)
Single mutant: F1076L	95% (95/100)	93%(66/71)	100% (29/29)
Double mutant: N1010S, F1076L	1% (1/100)	1.4% (1/71)	0% (0/29)
Triple mutant: L953F, N1010S, F1076L	1% (1/100)	1.4% (1/71)	0% (0/29)

### STR Genotyping

A total of 71/100 (71%) ‘pass’ samples were successfully genotyped at 5 or more of the 9 loci and were included in the population genetic analyses (Table 1). The pass samples comprised 57 autochthonous, 11 imported (10 imported and 1 putatively imported case) and 3 cases with unknown importation status. Genotyping data for the 71 pass samples are presented in the Supplementary Material (S3 Table). All 9 loci exhibited < 10% genotyping failures in the 71 pass samples (S4 Table). The 9 loci were all polymorphic in each of the patient nationality groups, although moderate variation was observed in diversity, with *H*_E_ values ranging from 0.57 at MS16 to 0.77 at MS8 (S4 Table).

### Relatedness

**Fig 2:**
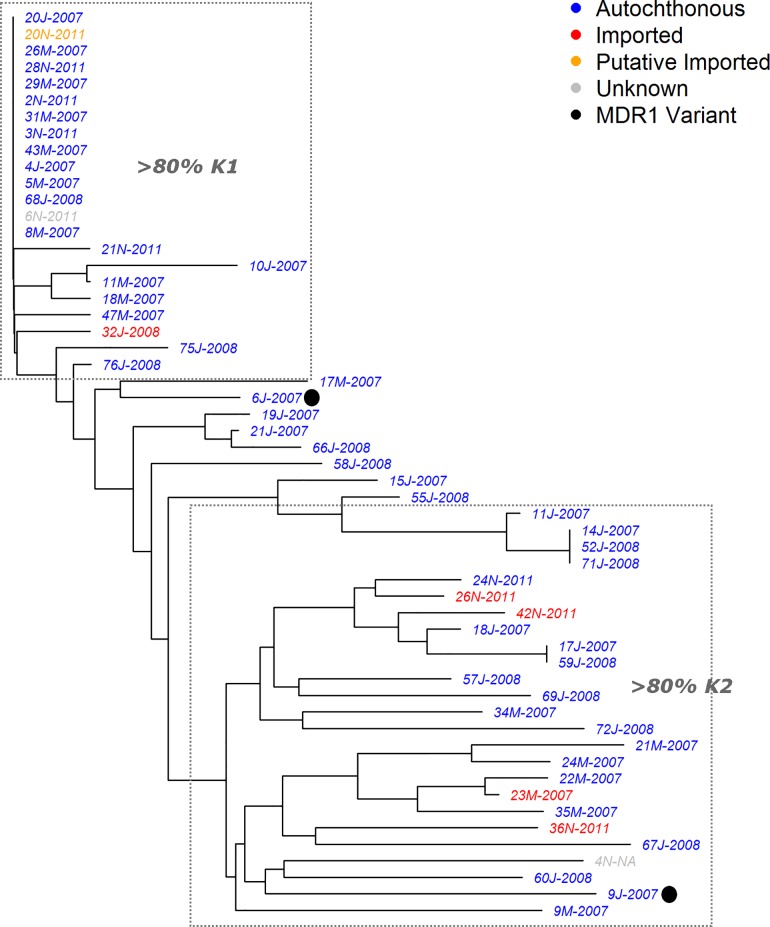
Neighbour-joining tree illustrating the genetic relatedness between the *P*. *vivax* isolates across 9 loci. Only isolates with complete genotyping data across all 9 loci are presented. The two isolates highlighted with black dots, 6J and 9J, exhibited differing *pvmdr1* multi-locus genotypes from the other isolates: L953-Y976-N1010-F0176 in 6J and 9J versus L953-Y976-N1010-L1076 in the other isolates. The dotted grey outlines illustrate the isolates with high ancestry to *K*1 and *K*2 at *K* = 2.

Neighbour-joining analysis of 55 isolates with no missing genotype data across the 9 loci highlighted a cluster of 14 isolates with identical MLGs (Fig 2). The identical isolates had a broad range of sources, comprising autochthonous and imported cases from Bandar Lengeh, Jask and Minab counties, presenting at the clinic between 2007 and 2011. Amongst the other 41 isolates included in the neighbour-joining analysis, only one cluster of infections (14J, 52J and 71J) displayed identical multi-locus genotypes (MLGs). The imported cases did not demonstrate any notable genetic separation from the autochthonous cases. Two of the six isolates with *Pvmdr1* mutations, 6J and 9J, had complete data across the 9 loci enabling inclusion in the neighbour-joining analysis. The two isolates, both of which carried F1076 mutations and which were sampled one day apart, presented distinct MLGs from one another as well as from the other isolates included in the analysis (Fig 2).

Mantel analysis indicated that there was no evidence of significant correlation between the distance in sampling date and the proportion of alleles shared between infections (Mantel *r*-test, *r* = -0.11, *P* = 0.942).

### Population Structure

**Fig 3:**
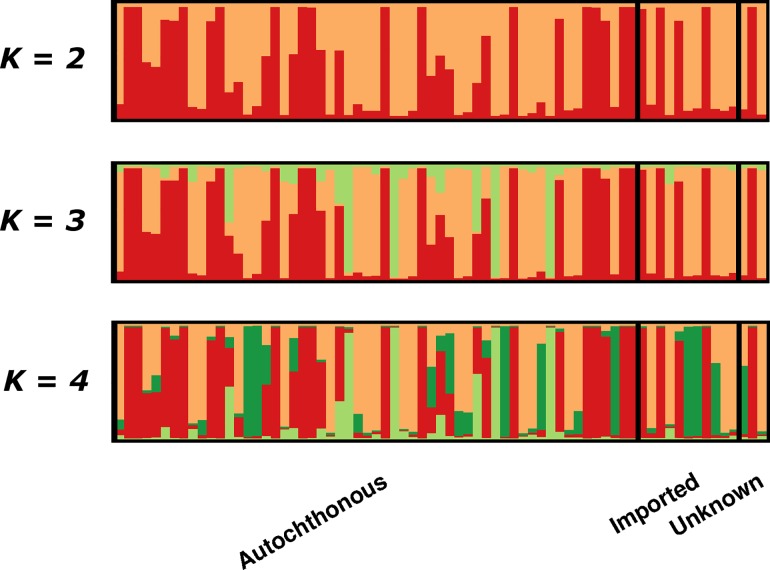
Population structure. Bar plots illustrating the population structure at *K* = 2, *K* = 3 and *K* = 4. Each vertical bar represents an individual sample and each colour represents one of the *K* clusters (sub-populations) defined by STRUCTURE. For each sample, the predicted ancestry to each of the *K* sub-populations is represented by the colour-coded bars. *K*1 = red, *K*2 = orange, *K*3 = light green and *K*4 = dark green. Samples are ordered by date of collection (oldest to newest) within each of the Autochthonous, Imported and Unknown sample groups.

Assessment of population structure using STRUCTURE software with *delta K* analysis inferred between 2 (*ΔK* = 99) and 4 (*ΔK* = 130) sub-populations (S1 Fig). As illustrated in Fig 3, at *K* = 2, a total of 24 (34%) isolates displayed high ancestry (>80%) to the *K*1 (red) sub-population, including the 14 isolates with identical 9 locus MLGs; 34 (48%) isolates displayed high ancestry to the *K*2 (orange) sub-population; and the remaining 13 (18%) isolates displayed moderately mixed ancestry to both sub-groups. At *K* = 4, the 24 (34%) aforementioned isolates maintained high ancestry to *K*1; 17 (24%) isolates maintained high ancestry to *K*2; 4 (6%) isolates displayed high ancestry to *K*3 (light green); 6 (8%) isolates displayed high ancestry to *K*4 (dark green); and 16 (23%) isolates displayed moderately mixed ancestry to two or more sub-populations. At both *K* = 2 and *K* = 4, isolates belonging to the predominant sub-populations did not demonstrate any notable differentiation between the autochthonous and imported groups, or any notable clustering by date of collection.

### Infection Complexity and Population Diversity

A summary of polyclonality, MOI and population diversity is presented in Table 3 for several population groups comparing population structure in autochthonous isolates and those imported. Across all autochthonous *P*. *vivax* infections, expected heterozygosity (*H*_E_) was 0.78, with 65% (37/57) of infections polyclonal (mean MOI = 1.98; median MOI = 2.0; range 1–5 clones). Excluding the 12 autochthonous isolates with identical MLGs, increased diversity (*H*_E_ = 0.75), prevalence of polyclonal infections (71%, 32/45) and complexity of infection as measured by the mean MOI (mean MOI = 2.09; median MOI = 2.0; range 1–5 clones). In accordance, when autochthonous/imported status was not considered and samples were grouped by majority ancestry to *K*1 or *K*2 at *K* = 2, the *K*1 sub-population exhibited lower population diversity than *K*2 (*H*_E_ = 0.06 vs 0.82), fewer polyclonal infections (50% vs 65%) and lower MOI (mean MOI = 1.58 vs 1.97; median MOI = 1.5 vs 2.0; range = 1–5 vs 1–4).

**Table 3:** Complexity of infection and population diversity.

Group	% Polyclonal infections (No. polyclonal/Total)	MOI (mean, median, range)	Expected heterozygosity (mean *H*_E_ ± SE, range)
**Autochthonous**	65% (37/57)	1.98, 2 (1–5)	0.669 ± 0.02 (0.569–0.765)
**Autochthonous subgroup** ^**1**^	71% (32/45)	2.09, 2 (1–5)	0.750 ± 0.02 (0.673–0.852)
***K*1**^**2**^	50% (12/24)	1.58, 1.5 (1–4)	0.056 ± 0.02 (0.000–0.163)
***K*2**^**3**^	65% (22/34)	1.97, 2 (1–5)	0.815 ± 0.02 (0.720–0.886)
**All**	62% (44/71)	1.93, 2 (1–5)	0.668 ± 0.02 (0.573–0.766)

^1^ Autochthonous sub-group excluding 12 infections with identical MLGs.

^2^ Isolates with > = 80% ancestry to *K*1 at *K* = 2.

^3^ Isolates with > = 80% ancestry to *K*2 at *K* = 2.

### Linkage Disequilibrium

A summary of LD values, as measured by the Index of Association (*I*_A_^S^), for samples grouped by autochthonous versus imported origin, and population structure, is presented in Table 4. High LD was observed amongst the 47 autochthonous *P*. *vivax* samples with 9-locus MLGs (*I*_A_^S^ = 0.343, *P*<0.01). Assessment of the 33 unique MLGs in this group revealed lower LD but remained moderately high and significant (*I*_A_^S^ = 0.158, *P*<0.01). Exclusion of the 12 identical MLGs in the autochthonous group, also resulted in lower LD but again remained significant when all 35 isolates were included (*I*_A_^S^ = 0.166, *P*<0.01) and when the 32 unique MLGs were considered (*I*_A_^S^ = 0.141, *P*<0.01). Owing to insufficient marker diversity amongst the isolates with high ancestry to *K*1 (S4 Table), and subsequent confounding effects on the Index of Association (*I*_A_^S^), LD analysis could not be undertaken in this group. In the *K*2 group, LD was high amongst the 25 isolates with 9-locus MLGs (*I*_A_^S^ = 0.206, *P*<0.01), declining slightly but remaining significant on assessment of the 22 unique MLGs in this group (*I*_A_^S^ = 0.136, *P*<0.01).

**Table 4:** Linkage disequilibrium.

Group	LD in all isolates ^1^, *I*_A_^S^ (*n*)	LD in unique MLGs ^2^, *I*_A_^S^ (*n*)
**Autochthonous**	0.343 (47) *	0.158 (33) *
**Autochthonous subgroup** ^**3**^	0.166 (35) *	0.141 (32) *
***K*2** ^**4**^	0.206 (25) *	0.136 (22) *
**All**	0.332 (55) *	0.143 (39) *

*I*_A_^S^ = index of association.

* *p* ≤ 0.01

^**1**^Only samples with no missing data at all 9 loci are included in the analyses

^2^ Unique set of multi-locus genotypes.

^3^ Autochthonous sub-group excluding 12 infections with identical MLGs.

^4^ Isolates with > = 80% ancestry to *K*2 at *K* = 2.

## Discussion

This study presents the first investigation of the genetic diversity and structure of *P*. *vivax* isolates in southern Iran using neutral STR markers, and the first assessment of *pvmdr1* polymorphism in the region. The study demonstrated complex population genetic patterns, revealing notable population sub-structure likely reflecting composite dynamics of local and imported transmission. Limited variation was observed in the *pvmdr1* gene, with the large majority of infections carrying single F1076L mutations on a wildtype Y976 background.

Previous studies using surface antigen markers have provided important insights into the local diversity of *P*. *vivax* in Iran, demonstrating lower diversity in isolates from the north versus south-eastern provinces [24, 25]. These findings appeared to reflect a combination of factors including the longer transmission period in the tropical south-eastern provinces relative to the temperate north. Furthermore, the northern *P*. *vivax* population, which had been malaria-free for approximately 20 years prior to re-introduction in 1994, appears to have re-emerged as a founder population from Azerbaijan and Armenia [3, 5]. In contrast, malaria has remained endemic at low levels in the south-eastern provinces, but these areas have reported large numbers of imported cases resulting from cross-border exchanges with Afghanistan and Pakistan [3–6]. The current study focused on malaria in Hormozgan Province in south-eastern Iran, providing results that complement the studies of Zakeri and colleagues by investigating the local *P*. *vivax* diversity using a different class of markers. The application of STR markers enabled investigation of local parasite transmission patterns without the potential constraint of host immunity-related selective pressures, and more comprehensive assessment of infection complexity.

STR-based analysis of the parasite population structure demonstrated clear substructure with evidence of at least two major sub-populations, defined here as *K*1 and *K*2. The *K*2 sub-population displayed greater complexity than the *K*1 sub-population, demonstrating evidence of internal sub-structure. Over 80% of isolates displayed high ancestry to either the *K*1 or *K*2 subpopulation. The two sub-populations displayed markedly different patterns of diversity suggesting that they might reflect different reservoirs of infection, with differing transmission dynamics.

The *K*1 sub-population exhibited very low diversity (*H*_E_ = 0.06) owing in large part to a cluster (*n* = 14) of infections with identical MLGs. A similar pattern reported in the low endemic setting of Sabah, Malaysia, was attributed to epidemic expansions [42]. However, in contrast to Sabah, the identical MLGs observed in Iran were sourced across multiple years and districts, suggesting moderately stable transmission. Cumulative data from different endemic settings indicates that transmission intensity and geographical isolation are important determinants shaping diversity and structure in *P*. *vivax* populations [23, 42–53]. In this respect, the trends observed here infer that the *K*1 sub-population may reflect a low intensity and moderately isolated reservoir of infection. One possibility is that the *K*1 infections have been introduced from northern Iran. Indeed, long latency phenotypes typical of temperate *P*. *vivax* strains may have facilitated the long-term persistence of these infections over the years. Alternatively, the *K*1 sub-population may represent autochthonous southeastern *P*. *vivax* isolates, with the highly differentiated *K*2 sub-population attributable to internationally introduced strains. Indeed, bottleneck effects on the shrinking residual population of *P*. *vivax* infections in the southeastern provinces may have greatly reduced local diversity. Assessment of the genotypes present in the northern *P*. *vivax* population using the APMEN STR markers is needed to add further insight into the likely origin of the *K*1 infections.

In marked contrast to the *K*1 sub-population, high diversity was observed in *K*2 (*H*_E_ = 0.82). Previous studies using the APMEN markers have demonstrated equivocally high diversity (*H*_E_ > 0.8) in a range of endemic settings including stable endemic settings in Indonesia [50] and Ethiopia [44], and the contrastingly low endemic, pre-elimination setting of Bhutan [53]. Similar to the situation in Iran, malaria elimination efforts in Bhutan have been undermined by cross-border malaria. As has been reported in Sri Lanka [46], a high influx of imported and introduced cases was thus suspected to sustain the observed diversity in Bhutan. The high diversity observed in *K*2 might reflect a similar dynamic of imported and introduced cases from neighboring Afghanistan and Pakistan. Indeed, in 2012, 46% of all malaria cases were imported [28]. Furthermore, a previous STR-based study of *P*. *vivax* diversity in populations from Afghanistan and Pakistan revealed extensive diversity (*H*_E_ = 0.98) [54].

In accordance with the high population diversity, the *K*2 sub-population comprised a high prevalence of polyclonal infections (65%). In comparison to other studies applying the same markers and methodologies for calling minor alleles, these levels are most comparable to those observed in moderate to high transmission settings in Indonesia [50] and Ethiopia [44]. In *P*. *falciparum* populations, the prevalence of polyclonal infections generally increases with increasing transmission intensity as a result of super-infection [33]. The high complexity of infection in Hormozgan Province is at odds with the low reported malaria incidence, and thus might reflect the contribution of complex infections introduced from Afghanistan and Pakistan, where endemicity is higher. Alternatively, the high complexity of infection might reflect heterogeneity in local transmission, with superinfections arising at local transmission ‘hot-spots’ or amongst ‘hot-pops’. Furthermore, whilst the number of reported clinical cases of malaria in Iran is low, the burden of asymptomatic and sub-patent infections remains unclear and could present a substantial reservoir. Indeed, the latter two possibilities could also explain the moderate proportion of polyclonal infections (50%) in the *K*1 sub-population. Comprehensive investigation of the burden of asymptomatic and sub-patent infections in Iran using molecular diagnostic methods such as new ultrasensitive PCR technologies [55, 56] would be highly informative for the NMCP.

Linkage disequilibrium (LD) could not be assessed in the *K*1 sub-population owing to the low allelic diversity across the markers in this group. In the *K*2 sub-population, a moderately high *I*_A_^S^ of 0.2 was observed, with no evidence of epidemic transmission dynamics. Relative to other studies applying the same markers, the *K*2 sub-population was most comparable to the meso-endemic setting of West Timor (*I*_A_^S^ = 0.19) [50], and to Bhutan (*I*_A_^S^ = 0.16) [53]. Over 60% of the infections in the Bhutanese study were imported from India, but epidemiological and molecular evidence of local transmission was also demonstrated; the resulting LD was postulated to reflect the composite of local inbreeding and imported infection transmission dynamics. The LD in the *K*2 sub-population might reflect a similar composition.

Our study was limited by the low number of imported cases (*n* = 10 pass samples), which prevented comprehensive analysis of the genetic differentiation between autochthonous and imported cases. The available evidence, based on neighbor-joining and STRUCTURE analysis, did not reveal any distinct separation between imported and autochthonous infections, inferring that frequent *P*. *vivax* gene flow between Iran, Afghanistan and Pakistan has reduced the differentiation between these populations. However, relapses from long-latency *P*. *vivax* infections may lead to incorrect classifications of parasite origin.

Imported infections are a major public health threat, presenting a parasite reservoir amenable to ongoing transmission and potentially introducing drug resistant strains from other locations. The current study presents the first assessment in Iran of the molecular epidemiology of the *pvmdr1* gene—a putative modulator of CQ resistance [16]. The polymorphism at codon F1076L of *pvmdr1* was present in almost all (97%) isolates, whereas the Y976F mutation, common in SE Asia was present in none of the isolates. Three *pvmdr1* mutant alleles were identified: F1076L alone, N1010S-F1076L and L953F-F1076L-N1010S. Overall 3% of isolates had wild-type pvmdr1 genotype, 95% had the single-mutant (F1076L), 1% the double-mutant (N1010-F1076L) and 1% the triple-mutant genotype (L953F-N1010S-F1076L). In accordance with these findings, a study in neighboring Pakistan also observed that all isolates were wild type at position Y976F and almost all (98%) carried the F1076L mutant.

On the island of Papua, CQ resistant *P*. *vivax* is highly prevalent, and the Y976F *pvmdr1* mutation has almost reached fixation [16, 57]. Conversely, in Thailand where *P*. *vivax* is mostly CQ sensitive, the prevalence of the Y976F polymorphism is at low levels [16]. Both 976 polymorphism and gene amplification of *pvmdr1* were correlated with reduced *ex vivo* susceptibility of *P*. *vivax* to CQ and mefloquine respectively [16, 58]. These studies have led to speculation that the 976 codon may contribute to CQR *P*. vivax [17]. However, since CQ resistance can occur in isolates with wild type *pvmdr1* it is highly likely that multigenic loci are responsible for CQR in *P*. *vivax*, and that the 976 loci is at best only a minor determinant [14]. Furthermore, the clinical relevance of *pvmdr1* polymorphism has yet to be confirmed. CQ remains an efficacious drug for the treatment of *vivax* malaria in southeast Iran [13], with no reports of CQ resistance. Hence the mutant alleles could represent geographical variation in *P*. *vivax* or the natural occurrence of this polymorphism in Iran.

## Conclusions

STR genotyping demonstrated two *P*. *vivax* sub-populations in Hormozgan Province, one with diversity consistent with low transmission and the other more consistent with higher transmission. These findings suggest that a combination of local transmission and cross-border malaria from higher transmission regions may shape the genetic make-up of the *P*. *vivax* population in south-eastern Iran. With the accumulation of CQ resistance across the vivax-endemic world, the risk that imported/introduced cases could introduce drug resistant strains justifies continued surveillance of the *P*. *vivax* population in Iran.

## Supporting Information

S1 Fig *Delta K* assessment of STRUCTURE output on the 71 successfully genotyped *P*. *vivax* isolates. (TIFF)

S1 Table Epidemiological data on *P*. *vivax* in Hormozgan Province in 2012. Data was provided by Dr Reza Safari, Deputy of Health, Hormozgan University of Medical Sciences, Bandar Abbas, Iran.
(DOCX)

S2 Table Genotyping primer details. (DOCX)

S3 Table Genotype calls and multiplicity of infection for each of the 71 successfully genotyped *P*. *vivax* isolates. (CSV)

S4 Table Marker diversity and genotyping success rates in the pass samples. Markers are listed in order of highest to lowest expected heterozygosity across all samples.
(DOCX)
