# Supplementary figures and images for: Molecular Epidemiology of P. vivax in Iran: High Diversity and Complex Sub-Structure Using Neutral Markers, but No Evidence of Y976F Mutation at pvmdr1
Source: PLoS One. 2016 Nov 9;11(11):e0166124. doi: 10.1371/journal.pone.0166124 (PMC5102416; doi:10.1371/journal.pone.0166124)

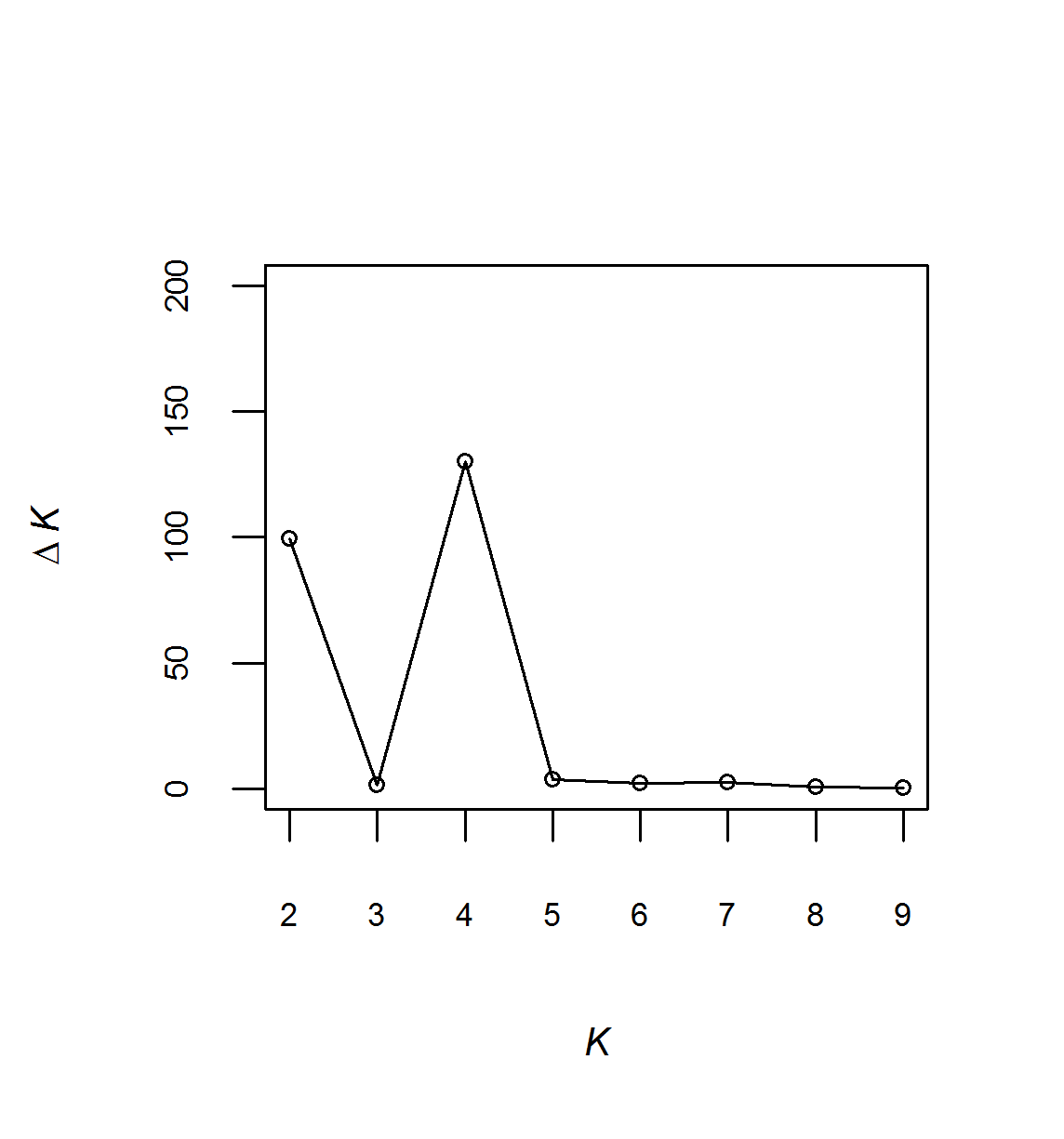

Supplement: S1 Fig — (TIFF) [file pone.0166124.s001.tiff]
