# Supplementary material for: Molecular Epidemiology of P. vivax in Iran: High Diversity and Complex Sub-Structure Using Neutral Markers, but No Evidence of Y976F Mutation at pvmdr1
Source: PLoS One. 2016 Nov 9;11(11):e0166124. doi: 10.1371/journal.pone.0166124 (PMC5102416; doi:10.1371/journal.pone.0166124)
Supplement: S1 Table — Data was provided by Dr Reza Safari, Deputy of Health, Hormozgan University of Medical Sciences, Bandar Abbas, Iran. (DOCX) [file pone.0166124.s002.docx]

**S1 Table. Epidemiological data on *P. vivax* in Hormozgan Province in 2012**

| **District** | **No. slides prepared** | **No. positive cases** | **^1^ API** | **^2^ SPR** | **^3^ ABER** |
| --- | --- | --- | --- | --- | --- |
| Bandar-e-lengeh | 3170 | 24 | 0.2 | 0.8 | 3.1 |
| Rudan | 4199 | 0 | 0 | 0 | 3.4 |
| Minab | 2944 | 11 | 0.05 | 0.4 | 1.2 |
| Jask | 7212 | 37 | 0.7 | 0.5 | 13.8 |
| Qeshm | 8215 | 20 | 0.2 | 0.2 | 6.7 |

Data was provided by Dr Reza Safari, Deputy of Health, Hormozgan University of Medical Sciences, Bandar Abbas, Iran

**^1^**Annual Parasite Incidence (No. reported cases per 1000 population)

^2^ Slide Positivity Rate (%)

^3^ Annual Blood Examination Rate (%)
