# Supplementary material for: Molecular Epidemiology of P. vivax in Iran: High Diversity and Complex Sub-Structure Using Neutral Markers, but No Evidence of Y976F Mutation at pvmdr1
Source: PLoS One. 2016 Nov 9;11(11):e0166124. doi: 10.1371/journal.pone.0166124 (PMC5102416; doi:10.1371/journal.pone.0166124)
Supplement: S2 Table — (DOCX) [file pone.0166124.s003.docx]

**S2 Table. STR Genotyping Primers.**

| **Marker** | **Primer name** | **Sequence 5' - 3'** |
| --- | --- | --- |
| MS1 | Primary Forward | CATCTCGACATGTCGACGTAG |
|  | Nest Forward | **6FAM**-TCAACTGTTGGAAGGGCAAT |
|  | Reverse | ctgtcttTTGCTGCGTTTTTGTTTCTG |
| MS8 | Primary Forward | AAACGTAAAACCTTTGGCGG |
|  | Nest Forward | **VIC**-AGAGGAGGCAGAAATGCAGA |
|  | Reverse | ctgtcttAGCCCCTTTGCGTTCTTTAT |
| MS10 | Primary Forward | AGGACCAAACGGAGGACATG |
|  | Nest Forward | **PET**-TTATCCCTGCTGGATGTGAA |
|  | Reverse | ctgtcttTCCTTCAGGTGGGACTTGTT |
| MS5 | Primary Forward | TTCGGCTGGTTTCCAATTAGG |
|  | Nest Forward | **NED**-CGTCCTCTATCGCGTACACA |
|  | Reverse | ctgtcttAAAGGGAGAGGAGCGAAAAC |
| MS12 | Primary Forward | AACGTTTCCTTGCCCACTTG |
|  | Nest Forward | **6FAM**-AATGCGCATCCTATGTCTCC |
|  | Reverse | ctgtcttCTGCTGTTGTTGTTGCTGCT |
| MS20 | Primary Forward | CAAGGTGCGATGGAAGATTGG |
|  | Nest Forward | **VIC**-GCACAACAAATGCAAGATCC |
|  | Reverse | ctgtcttGTGGCAGTGGCTCATCTTCT |
| MS16 | Primary Forward | TTCCTGATGACAATTTCGACGG |
|  | Primary Reverse | TCTCTTCCCATTTGAGCATCGC |
|  | Nest Forward | **PET**-CTTGTTGTGGTTGTTGATGGTG |
|  | Nest Reverse | **ABDTAIL**-AGTACGTCAACCATGTGGGTAG |
| Pv3.27 | Primary Forward | TTTTTCAACTTGCTGCCCCCTG |
|  | Nest Forward | **6FAM**-GGACATTCCAAATGTATGTGCAGTCG |
|  | Reverse | **ABDTAIL**-CGTCATCGTCATTGCTCTGGAG |
| msp1F3 | Primary Forward | GGAGAACATAAGCTACCTGTCC |
|  | Primary Reverse | GTTGTTACTTGGTCTTCCTCCC |
|  | Nest Forward | **VIC**-CAAGCCTACCAAGAATTGATCCCCAA |
|  | Nest Reverse | **ABDTAIL**-ATTACTTTGTCGTAGTCCTCGGCGTAGTCC |
