# Supplementary material for: Molecular Epidemiology of P. vivax in Iran: High Diversity and Complex Sub-Structure Using Neutral Markers, but No Evidence of Y976F Mutation at pvmdr1
Source: PLoS One. 2016 Nov 9;11(11):e0166124. doi: 10.1371/journal.pone.0166124 (PMC5102416; doi:10.1371/journal.pone.0166124)
Supplement: S4 Table — Markers are listed in order of highest to lowest expected heterozygosity across all samples. (DOCX) [file pone.0166124.s005.docx]

**S4 Table. Marker diversity and genotyping success rates in the pass samples.**

| **Marker** | **Endemicity correlation [**[18](#_ENREF_18)**]** | **% Genotyping failures** |  | | **Expected Heterozygosity** | |  | | |
| --- | --- | --- | --- | --- | --- | --- | --- | --- | --- |
|  |  |  | **Autochthonous** | **Imported** | | ***K*1 ^1^** | | ***K*2 ^2^** | **All** |
| **MS8** | Excess diversity | 7.0% (5/71) | 0.765 | 0.822 | | 0.083 | | 0.857 | 0.766 |
| **MS20** | Balanced | 9.9% (7/71) | 0.697 | 0.722 | | 0.000 | | 0.841 | 0.721 |
| **msp1f3** | Putative selection | 2.8% (2/71) | 0.712 | 0.756 | | 0.083 | | 0.786 | 0.712 |
| **MS10** | Balanced | 5.6% (4/71) | 0.688 | 0.750 | | 0.087 | | 0.837 | 0.700 |
| **MS1** | Balanced | 0% (0/71) | 0.677 | 0.691 | | 0.000 | | 0.775 | 0.688 |
| **MS5** | Balanced | 1.4% (1/71) | 0.606 | 0.778 | | 0.087 | | 0.720 | 0.648 |
| **pv3.27** | Excess diversity | 8.5% (6/71) | 0.650 | 0.533 | | 0.000 | | 0.886 | 0.627 |
| **MS12** | Balanced | 2.8% (2/71) | 0.569 | 0.511 | | 0.163 | | 0.806 | 0.575 |
| **MS16** | Excess diversity | 8.5% (6/71) | 0.593 | 0.583 | | 0.000 | | 0.823 | 0.573 |
|  |  |  | 0.662 ± 0.02 | 0.683 ± 0.04 | | 0.087 ± 0.02 | | 0.815 ± 0.02 | 0.668 ± 0.02 |

Markers listed in order of highest to lowest expected heterozygosity across all samples.

^1^ Isolates with >=80% ancestry to *K*1 at *K*=2 (n=24).

^2^ Isolates with >=80% ancestry to *K*2 at *K*=2 (n=34).
